# Application of PCR-Based Tools to Explore *Strongyloides* Infection in People in Parts of Northern Australia

**DOI:** 10.3390/tropicalmed2040062

**Published:** 2017-12-08

**Authors:** Gemma J. Robertson, Anson V. Koehler, Robin B. Gasser, Matthew Watts, Robert Norton, Richard S. Bradbury

**Affiliations:** 1 Public and Environmental Health, Forensic and Scientific Services, Health Support Queensland, Brisbane, QLD 4108, Australia; 2 Department of Veterinary Biosciences, The University of Melbourne, Melbourne, VIC 3053, Australia; anson.koehler@unimelb.edu.au (A.V.K.); robinbg@unimelb.edu.au (R.B.G.); 3 Centre for Infectious Diseases and Microbiology, Institute for Clinical Pathology and Medical Research NSW Health Pathology, Westmead Hospital, Westmead, NSW 2145, Australia; matthew.watts@health.nsw.gov.au; 4 Marie Bashir Institute for Infectious Diseases and Biosecurity, University of Sydney, Westmead, NSW 2145, Australia; 5 Pathology Queensland, The Townsville Hospital, Townsville, QLD 4814, Australia; Robert.Norton@health.qld.gov.au; 6 School of Health, Medical and Applied Sciences, Central Queensland University, North Rockhampton, QLD 4700, Australia; r.bradbury@cqu.edu.au

**Keywords:** strongyloidiasis, *Strongyloides stercoralis*, Australia, PCR, SSCP

## Abstract

Strongyloidiasis, which is caused by infection with the nematode *Strongyloides stercoralis*, is endemic to areas of northern Australia. Diagnosis in this region remains difficult due to the distances between endemic communities and diagnostic laboratories, leading to lengthy delays in stool processing for microscopy and culture. PCR represents a viable solution to this difficulty, having potential for high sensitivity detection of *S. stercoralis*, even in older, unpreserved faecal samples. We prospectively collected 695 faecal specimens that were submitted to The Townsville Hospital Microbiology Laboratory from the North Queensland region for routine parasitological examination, and subjected them to a *Strongyloides* sp. real-time (q)PCR. Results were confirmed with a novel nested conventional PCR assay targeting the 18S rRNA gene, followed by single-strand conformation polymorphism analysis (SSCP). Of the 695 specimens tested, *S. stercoralis* was detected in three specimens (0.4%) by classical parasitological methods (direct microscopy and formyl-ether acetate concentration), whereas 42 positives were detected by qPCR (6.0%). Conventional PCR confirmed the real-time PCR results in 24 of the samples (3.5%). Several apparent false-positive results occurred at higher cycle times (*C*_t_) in the qPCR. Use of real-time PCR in these populations is promising for the enhanced detection of disease and to support eradication efforts.

## 1. Introduction

Strongyloidiasis is an intestinal helminthic disease of humans with protean clinical presentations, including acute enteritis, chronic asymptomatic infection, or a potentially fatal hyperinfection syndrome [1]. The causative agent, *Strongyloides stercoralis*, is a nematode that is endemic to tropical and sub-tropical regions of the world. The parasitic life cycle includes an auto-infective component, and, therefore, without the appropriate treatment, infection with this nematode can persist for decades [1]. Estimates indicate that 30–370 million people are infected with this parasite worldwide [2]. This variation in estimated burden of infection is attributable to a number of factors, the most important being the difficulty in achieving an accurate diagnosis of infection, particularly in people with low-burden, chronic strongyloidiasis [3,4]. 

There is a paucity of detailed information regarding the prevalence and distribution of autochthonous strongyloidiasis in Australia, and methodologies vary considerably among studies. Prevalences of 0.26% and 1.9% were recorded from surveys in north Western Australia in 1993 and 1997, using low-sensitivity diagnostic techniques (direct microscopy, ZnSO_4_ flotation, and Kato-Katz) [4,5]_._ In the Northern Territory, prevalences that were reported ranged from 7.2 to 59.6% (direct microscopy, concentration techniques, serology) [6,7,8,9]. This wide range in prevalence is reflective of the difficulties in diagnosis of this disease, with many traditional faecal microscopy methods having poor sensitivity for the detection of strongyloidiasis. The most comprehensive investigation published to date on strongyloidiasis in Australia was from Queensland [10], a state that includes the Tropical North. This study was conducted via the Aboriginal Health Program (AHP), which was established by the State Health Department in 1972. Single formalin-fixed stool samples that were collected from children in Aboriginal and Torres Strait Islander (ATSI) communities were sent to a central laboratory, where they underwent formol-ether concentration (FEC) and were examined by trained microscopists [10]. The overall annual prevalence ranged from 0 to 4.7% between 1972 and 1991, based on annual reports, with an average overall prevalence of 1.97% [10]. Two communities within the north-west region, Doomadgee and Mornington Island (Gununa), had a remarkably high prevalence (up to 26.0% and 27.5%, respectively). Based on results from the AHP data, strongyloidiasis was most prevalent in northern coastal areas of the state and was associated with areas of high rainfall, humidity, and temperature [10]. These data have not been updated for over 25 years, and the current prevalence of strongyloidiasis in Queensland is unknown. In each of these studies [4,5,6,7,8,9,10], the prevalence of infection is likely to be underestimated because of the low sensitivity of the diagnostic methods that were used [11,12,13]. Although comparisons of prevalence among geographic locations are unreliable due to the distinct methods that are employed among studies, strongyloidiasis is known to be common in residents of remote ATSI communities, north of the Tropic of Capricorn [14].

The Townsville Hospital Microbiology Laboratory (TTHML) services a large geographical area that is home to a diverse sociodemographic population, including a large ATSI population that lives in several small communities (including Doomadgee and Mornington Island) remote to the laboratory location. The majority of the population is concentrated within the major centre of Townsville, a regional city with a historically low prevalence of strongyloidiasis [10]. Despite the rate of detection of strongyloidiasis decreasing by ~40% since 2003 statewide (unpublished data), the methodologies that were utilised to detect these infections at TTHML do not offer confidence that the number of infections has reduced in real terms. Techniques such as agar plate culture and the Baermann sedimentation method [15], are the gold standard for the detection of infection with *S. stercoralis*. However, several factors been persistent barriers to their implementation in the Australian diagnostic laboratory setting. These factors include: long specimen transport times after collection in remote communities, potential exposure to extremes of temperature during transit, variable sensitivities of different diagnostic methodologies [15,16,17,18], intermittent shedding and uneven distribution of larvae in faeces [19], limited viability of larvae [20], subjectivity in the interpretation of morphological characteristics of larvae for differentiation from hookworms [1], limited resources and technical complexity of the assay, and the risk of infection to laboratory staff [21,22,23]. Modified concentration techniques (e.g., FLOTAC) [24] have emerged as alternatives to the gold standard (i.e., agar plate and Baermann methods), but the sensitivity and specificity of these methods vary considerably in the published literature [25,26,27,28]. The introduction of formalin-ether-acetate concentration (FEAC; Mini-Parasep kit, Apacor, Berkshire, UK) at TTHML in 2012 may have led to a reduction in the detection of infected patients; product information for this kit, as provided by the manufacturer, indicates that this system should not be used for the detection of *Strongyloides* larvae [29]. It is likely that this methodology concentrates out helminth larvae from the sediment, leading to false negatives. For these reasons, serological methods have been heavily relied upon for diagnosis and management of strongyloidiasis in Queensland, due to analyte stability [30], favourable sensitivity and specificity profiles [31], and their utility for monitoring the antibody response following treatment [32]. Nonetheless, the variations in sensitivity and specificity [33], and interassay variability [34], present limitations for serological techniques, particularly when they are used as the sole diagnostic tool. 

Molecular diagnostic methods offer an attractive solution to many of the problems that are inherent to the diagnosis of *Strongyloides* infection using faecal samples in northern Australian diagnostic laboratories. The most commonly used and widely validated molecular diagnostic test for the detection of strongyloidiasis worldwide is the real-time PCR that was developed by Jaco Verweij and co-workers [35], which uses a DNA region in the small subunit of the nuclear rRNA (18S) gene as a marker. A loop-mediated isothermal amplification method has also been developed, which shows major potential for implementation in areas with minimal laboratory infrastructure [36]. In the present study, we used the real-time method [35] in a diagnostic service laboratory in Queensland for the diagnosis of *Strongyloides* sp. infection in communities with a low apparent prevalence of strongyloidiasis, and then employed PCR-based mutation scanning [37] to assess levels of genetic variation within *Strongyloides* sp. from these communities. 

## 2. Materials and Methods

### 2.1. Ethics

Approval for specimen collection and analysis was given by The Townsville Hospital and Health Service Human Research Ethics Committee, reference number HREC/14/QTHS/4. 

### 2.2. Specimen Collection and Study Site 

**Figure 1:**
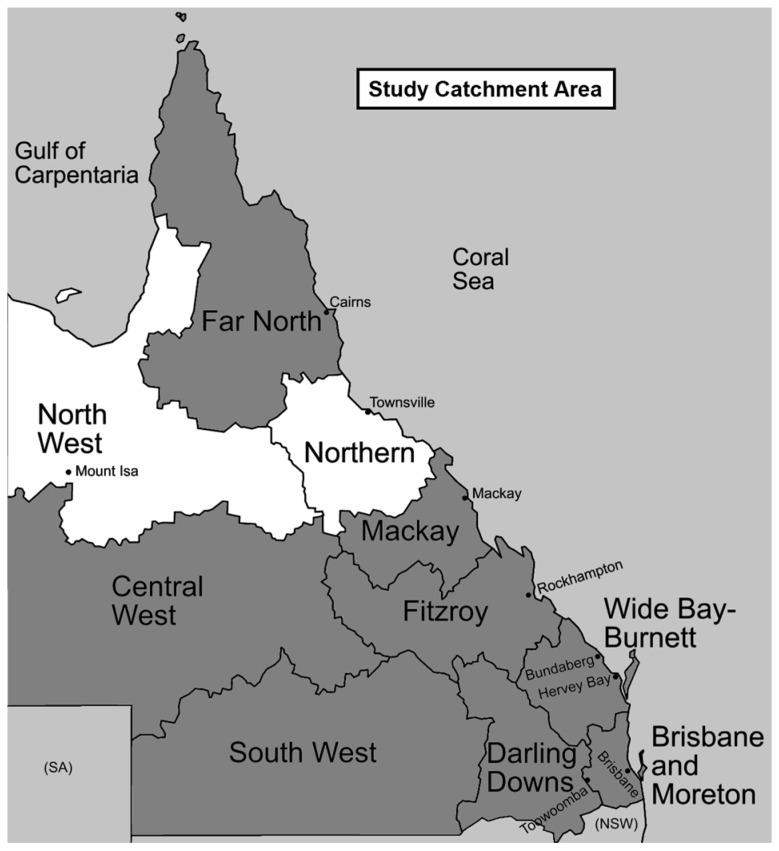
Fresh faecal specimens sampled for *Strongyloides* PCR were submitted from laboratories in the regions indicated. 90.7% of specimens received came from patients residing in these areas, and 95% of specimens positive by qPCR came from patients residing in these areas. Two positive specimens came from the Far North and Central West regions, and one specimen came from a small community (not shown) just over the western border of the North West region.

Stool specimens that were sent to TTHML for investigation of parasitic infections were collected over a one-year period (January–December 2014). Faecal specimens for parasitological examination are sent to TTHML from a large geographic area (478,446 km^2^), comprising the North West, Northern, and Mackay regions of Queensland (Figure 1). Across this area, the average daily temperature ranges from 17.3 °C to 30.2 °C, with an average rainfall of 702 mm per year [38]. The total human population of these regions is 431,300, with 224,700 (52.1%), 171,600 (39.8%), and 35,000 (8.1%) people residing in the Northern, Mackay, and North West regions, respectively. In the North West region, the proportion of the population that identify as ATSI is 21.7%, more than three times the proportion in the Northern and Mackay regions (7.1% and 4.1%, respectively). However, all of the regions have a higher proportion of ATSI residents as compared to the state as a whole, with Indigenous residents comprising 3.6% of the entire Queensland population [38]. 

Both fresh and formalin fixed faecal samples are received from the North West and Northern regions, while only fixed specimens (that are unsuitable for PCR testing) are received from the Mackay region. Specimens from the North West region are refrigerated during transport. A distinction was not made between inpatients and outpatients, due to the chronicity of *Strongyloides* infection. Specimens were aliquoted into 100% ethanol at a 1:5 dilution using approximately 1 g of stool and were stored at 4 °C. 

### 2.3. Microscopy

All of the specimens with a request for ova, cysts, and parasites (OCP) underwent direct microscopy and FEAC using the Mini-Parasep Faecal Parasite Concentrator (Apacor, Wokingham, UK). Briefly, 0.4 g of faeces was introduced to the Mini-Parasep mixing chamber, along with 3.3 mL of SAF and one drop of Triton-X-100. The tube was sealed with the filtration unit and thoroughly vortexed to emulsify the specimen. Tubes were left to stand for 30 min to complete the fixation process. The tube was then inverted and centrifuged at 500 *g* for 5 min, and the supernatant was discarded. A wet preparation of the sediment in both saline and Lugol’s iodine was examined for ova, cysts, and helminths at ×100 and ×400 under a light microscope, followed by the preparation of permanent smears using a modified iron-haematoxylin stain for examination at ×1000. Requests specifically asking for detection of a hookworm or *Strongyloides* infection were also processed using the Harada-Mori technique, as previously described [39]. The sediment was examined at ×400 under a light microscope for larvae. 

### 2.4. DNA Extraction

Ethanol-preserved faecal specimens were centrifuged at 10,000× *g* for 10 min and the supernatant discarded. Specimens were then resuspended in 1 mL of sterile saline and were left overnight at 4 °C. Following centrifugation at 10,000× *g* for 10 min, the supernatant was discarded, and DNA was extracted from the pellet using the PowerSoil DNA Isolation Kit (Mo Bio Laboratories, Carlsbad, CA, USA), according to the manufacturer’s instructions, and was stored at −80 °C. 

### 2.5. Real-Time PCR

This PCR assay was performed using primers (forward 5′-GAA TTC CAA GTA AAC GTA AGT CAT TAG C-3′, reverse 5′-TGC CTC TGG ATA TTG CTC AGT TC-3′) and probe (FAM-5′-ACA CAC CGG CCG TCG CTG C-3′-BHQ1) targeting a 101 bp region of the 18S rRNA gene (cf. GenBank accession number AF279916), as described by Verweij et al. [35], using a reaction volume of 25 μL [40]. The PCR mixture comprised 0.3 μM of each primer, 0.1 μM of probe, 5 mM MgCl_2_, 0.1 mg/mL bovine serum albumin (BSA) (Fisher Biotec, Australia), and 5 μL of DNA template in a final volume of 25 μL of PCR buffer (HotStarTaq Master Mix, QIAGEN, Hilden, Germany). PCR was performed on a Corbett Rotor-Gene 6000 (QIAGEN) under the following conditions: 15 min at 95 °C, followed by 45 cycles of 15 s at 95 °C, and 30 s at 60 °C. Detection and analysis of products was performed using Corbett Rotor Gene 6000 Series Software (QIAGEN, Hilden, Germany). Thresholds were determined at each run visually, with a cut-off cycle time (*C*_t_) of 40.

Specimens with amplification detected up to a *C*_t_ value of 40 had a repeat amplification that was performed to confirm the result. Duplicate results of *C*_t_ < 40 were considered as PCR positive, while those that failed to produce a product on repeat amplification were considered PCR equivocal. Real-time PCR results were compared to the results of microscopic testing (direct microscopy and FEAC) to determine sensitivity and specificity.

### 2.6. PCR-Based Single-Strand Conformation (SSCP) Analysis and Sequencing

All of the genomic DNA samples that were shown to be test-positive by real-time PCR were subjected to nested PCR utilising new pairs of primers designed to the 18S rRNA gene using Geneious [41] and Primer3 software [42]. The primer pair Strong155F (5′-TAA AGG AAT TGA CGG AAG GGC A-3′) and Strong578R (5′-TCC CAG TTA CGT AAT GTT TTC AAT GTT-3′) was used in the primary PCR to amplify a 423 bp region, and secondary primer pairs Strong198F (5′-GCT TAA TTT GAC TCA ACA CGG GAA-3′) and Strong492R (5′-CCC GGA CAT CTA AGG GCA TC-3′) were used to amplify a 294 bp region of the gene. 

**Figure 2:**
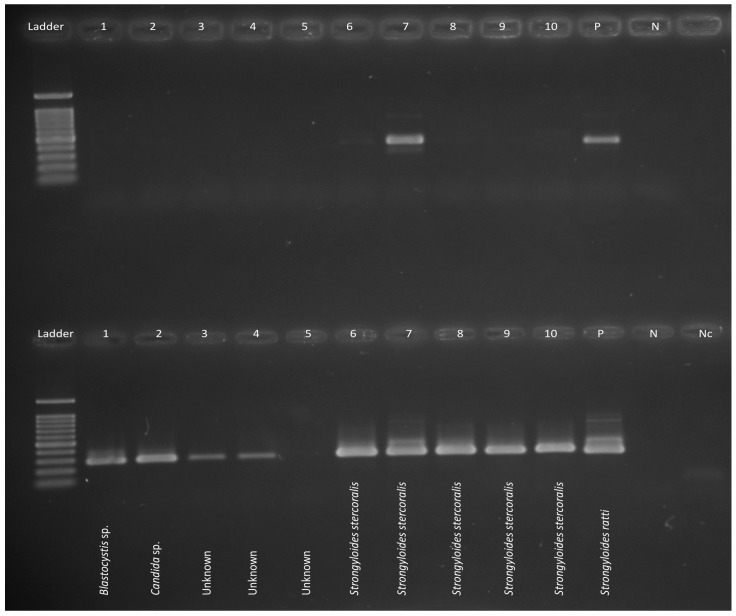
Conventional nested PCR testing a subset of samples, with primary PCR on the top row and secondary PCR on the bottom row. A 100 bp ladder (Promega) is used as a marker in the first lane. *Strongyloides ratti* was used as the positive control along with negative controls. The primers are non-specific therefore the single strand conformational polymorphism (SSCP) method was employed (see Figure 3).

Nested PCR was carried out in 50 μL containing 10 mM Tris-HCl (pH 8.4), 50 mM KCl (Promega, Madison, WI, USA), 3.0 mM of MgCl_2_, 200 μM of each deoxynucleotide triphosphate, 50 pmol of each primer, and 1 U of Go*Taq* (Promega) DNA polymerase. PCR reactions were performed on a Veriti thermal cycler (Applied Biosystems, Foster City, CA, USA) under the following conditions: initial denaturation at 94 °C/5 min, denaturation, annealing, and extension cycles of 30 s at 94 °C, 30 s at 64 °C, and 30 s at 72 °C for 35 cycles, followed by a final extension of 72 °C for 5 min. The quality, size, and intensity of individual PCR amplicons were assessed by electrophoresis (7 V/cm) in 1.5% agarose gels using TBE (65 mM Tris-HCl, 27 mM boric acid, 1 mM EDTA, pH 9; Bio-Rad, Hercules, CA, USA) as the buffer. Following electrophoresis, gels were stained with ethidium bromide and their size was estimated by comparison to PhiX174-*Hae*III (Promega, Madison, WI, USA) markers (Figure 2).

SSCP analysis [37] was used to scan for sequence variation within and among 18S amplicons. In brief, 1 μL of each secondary amplicon was mixed with 5 μL of DNA sequencing-stop solution (Promega) and 5 μL of H_2_O, heat-denatured at 94 °C/30 min, snap-cooled on a freeze-block (−20 °C), and then subjected to electrophoresis at 74 V at 7.4 °C (constant) for 16 h in a GMA Wide Mini S-2 × 25 gel in a SEA 2000 rig (Elchrom Scientific AG, Cham, Switzerland) using TAE buffer (40 mM Tris base, 20 mM acetic acid, 1.0 mM EDTA, Bio-Rad, USA). A control sample (extracted DNA of *S. ratti*) was included on each gel to ensure the reproducibility of profiles representing this sample among gels. Subsequently, 5 μL aliquots of individual amplicons representing all distinct electrophoretic profiles were treated with ExoSAP-IT^®^ (Affymetrix, Santa Clara, CA, USA), according to the manufacturer’s instructions, and then subjected to direct, automated sequencing (BigDye Terminator v.3.1 chemistry, Applied Biosystems, Foster City, CA, USA).

### 2.7. Eosinophil Counts and Strongyloides Serology

The most recent eosinophil counts (within three months) and *Strongyloides* serology (Bordier IVD EIA, Crissier, Switzerland; somatic antigens from larvae of *Strongyloides ratti*) results for each patient were extracted from the laboratory information system, where available. The reference ranges for *Strongyloides* serology are as follows: signal-to-cut off (S/CO) > 1.1 is considered positive; S/CO between 0.9–1.1 is considered equivocal, and S/CO < 0.9 is negative. The reference range for eosinophil count is dependent upon age and is reported using standard cut-offs that are produced by Pathology Queensland.

### 2.8. Statistical Analysis

The Student’s *t*-test (in Microsoft Excel version 15.0.1911.1000) was used to assess the statistical difference between *C*_t_ values; a *p*-value of < 0.05 was considered to be significant.

## 3. Results

### 3.1. Patient Demographics and Specimen Selection

Over the period January to December 2014, 1912 faecal specimens were submitted to TTHML for diagnosis of parasitic infection: 1080 (56.5%) originated from the Northern region laboratory, 463 (24.2%) from the Mackay region laboratory, and 369 (19.3%) from the North West region laboratory. Fresh faecal specimens accounted for 1449 of the total, and, of these, 706 (48.7%) were sampled for real-time PCR. Specimens were selected based on volume available for sampling, demographic spread, and resource constraints. Eleven specimens were misplaced; the final number of specimens that were able to undergo DNA extraction and PCR was 695 (48.0%). The average age of participants was 43.3 years (median 53 years, range one month to 97 years; 48.8% male, 51.2% female). A relative majority of samples came from patients aged more than 60 years (34.9%) (Figure 4). Thirty (4.2%) specimens were from patients residing in the Mackay region, 499 (70.6%) from patients residing in the Northern region, 141 (20.0%) from patients residing in the North West region, and 36 (5.1%) from patients residing in other areas (predominantly the Far North region) (Figure 1). 

### 3.2. Microscopy, Harada-Mori Culture and Real-Time PCR Results

Of the 695 faecal samples, five samples from three people were test-positive for larvae of *S. stercoralis* by microscopy, but no larvae were detected in any samples by Harada-Mori culture. By contrast, 42 samples were test-positive by real-time PCR, with a mean *C*_t_ value of 34.44 (median 36.34, geometric mean 33.97, range 18.77 to 39.33) (Table 1), and 12 samples were equivocal, with a mean *C*_t_ value of the initial positive result being 38.46 (median 38.37, geometric mean 38.44, range 36.32 to 39.96). By comparison to microscopy on FEAC samples, the sensitivity of the PCR was 100% (95% CI 47.82–100.00%), while specificity was 94.91% (95% CI 93.05–96.39%). It should be noted that calculations of sensitivity and specificity are not in comparison to a gold standard test, and will therefore overestimate sensitivity and underestimate specificity. Six samples were deemed low-level positives, with *C*_t_ values ranging from 40.28 to 42.13 on duplicate runs, and were included for further analysis. Twenty-two (36.7%) of the test-positive, equivocal, and low-level samples were from the North West region, and 35 (58.3%) were from the Northern region (Figure 1). One test-positive sample was from the Central West region, one equivocal specimen was from the Far North, and another equivocal specimen a short distance across the border into the Northern Territory. All five samples that were shown to contain *S. stercoralis* larvae by microscopy were test-positive by real-time PCR, with a mean *C*_t_ value of 25.61 (median 23.72, geometric mean 24.79, range 18.77 to 35.25). Numbers positive by microscopy were too few to allow for the statistical comparison of *C*_t_ values by the Student’s *t*-test.

### 3.3. PCR-SSCP Analysis and Sequencing

PCR-based mutation scanning was conducted on 60 samples that were test positive (*n* = 42), equivocal (*n* = 12), or low-level positive (*n* = 6) (Table 1). Twenty-five of the 42 positive samples (59.5%) and five of the 12 equivocal samples (41.7%) contained DNA sequences that were consistent with *S. stercoralis* by sequencing and SSCP. Ten of the positive samples, five of the equivocal samples, and one of the low-level positive samples demonstrated non-specific amplification by nested PCR. These were further characterised by sequencing as human gene (*n* = 5), *Blastocystis hominis* (*n* = 4), *Candida* sp. (*n* = 3), *Saccharomyces* sp. (*n* = 2), *Hymenolepis nana* (*n* = 1), or *Entamoeba coli* (*n* = 1).

**Figure 3:**
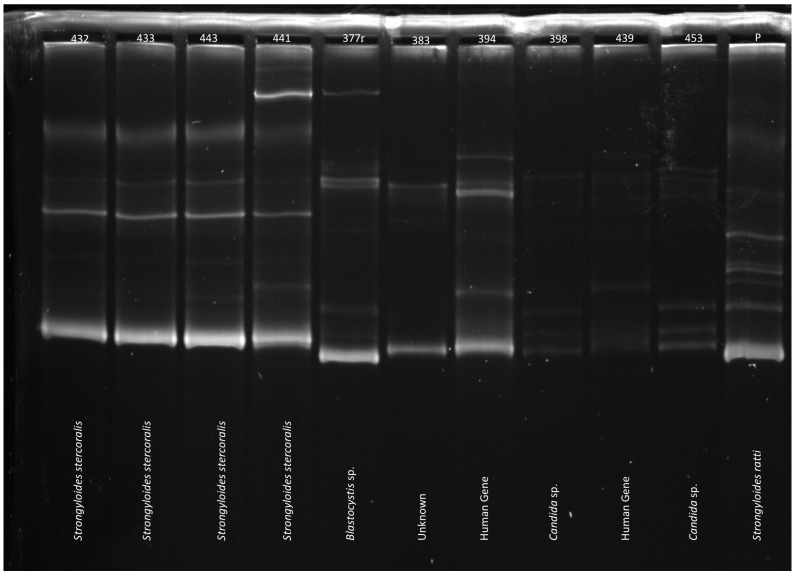
Sample single-strand conformational polymorphism (SSCP) gel, with text denoting results of sequencing. The banding profile for *Strongyloides stercoralis* is easily distinguishable from the other profiles which included *Blastocystis* sp., *Candida* sp. and human genes. Note prominent extra band in lane 441, which is most likely the result of conformers linked to sequence variability or background amplification.

**Figure 4:**
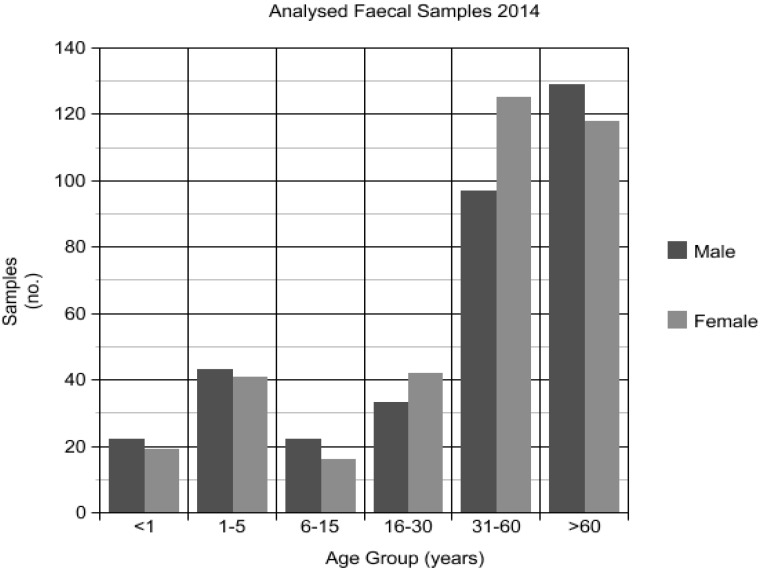
Demographics of patient faecal samples received at Townsville Hospital Microbiology Laboratory (TTHML) in 2014 and assessed by *Strongyloides* sp. qPCR.

The *S. stercoralis* sequences that were generated showed no sequence variability; however, two samples were found to have an extra band by SSCP analysis, which could represent conformers that are linked to sequence variability or background amplification (Figure 3). One sample came from a patient in the regional city of Townsville, while the other sample came from a patient in Charters Towers, approximately 150 km south-west. Further characterisation of the hypervariable region IV is required to determine the significance of these results. 

### 3.4. Comparison of Serology and Eosinophilia with qPCR Results

Twelve PCR test-positive and equivocal samples (20.0%), for which no *S. stercoralis* larvae were detected by microscopy, were from people who had eosinophilia and/or antibodies against *Strongyloides* (Table 1). These 12 samples had a mean *C*_t_ value of 34.17 (median 35.96, geometric mean 33.85, range 25.66 to 38.84). When the 11 PCR test-positive and equivocal samples for which no eosinophil count was available were excluded from the analysis, *C*_t_ values were significantly higher for those with eosinophilia than for those without by a Student’s *t*-test (*p* = 0.0135).

Sixteen patients that were included in this study had *Strongyloides* serology testing within the previous two years, 11 of which yielded positive titres. Only six of these serology-positive patients showed positive (*n* = 5) or equivocal (*n* = 1) results in the real-time PCR. All of these six had serology collected within three months of faecal specimen collection (range two days–three months). By nested PCR, four yielded *Strongyloides* sequences, whilst one yielded a sequence from *B. hominis*. One patient with positive serology and with equivocal real-time PCR yielded sequences for *Entamoeba coli* by nested PCR. Five of the 16 patients tested had returned positive serology in the previous two years, two on the same day as faecal specimen collection (range, one day–one year, nine months), but tested negative by real-time PCR. The treatment history of these patients is unknown.

Thirteen of those positive by real-time PCR also showed eosinophilia; nine of these yielded *Strongyloides* sequences by nested PCR, with the remaining four amplifying sequences of *B. hominis*, *H. nana*, human gene, and *Saccharomyces* sp. Only one sample with an elevated eosinophil count was equivocal by real-time PCR, which yielded a *Candida* sp. sequence on nested PCR. A single sample with equivocal results, but no eosinophilia, had a reactive *Strongyloides* serology titre.

## 4. Discussion

This study provides clear evidence of strongyloidiasis in North Queensland. The majority of PCR positive specimens (36.0%) came from the north-west region of Queensland, out of proportion to the number of samples that were submitted from that area (142, or 20.1%). This is consistent with the known epidemiology of the region and the very remote nature of the North West, with consequent difficulties in the delivery of health services, sanitation, and effective health education to the many of the small communities scattered throughout that region. 

The primary difficulty in determining positive and negative predictive values for *Strongyloides* PCR in this low prevalence population was the absence of a sensitive gold standard comparator. In an attempt to overcome this limitation, a sensitive, but not specific, nested PCR with subsequent SSCP and sequencing was performed. Previous studies of the efficacy of real-time PCR for the diagnosis of strongyloidiasis have either been performed in endemic, high-prevalence settings, or in patients with a high pre-test probability as a result of travel to an endemic region [34,35,43]. Such patient cohorts provide a high likelihood of true positive results, and thus a high positive predictive value for the test performed. The setting of a regional diagnostic laboratory in North Queensland offered a unique opportunity to determine the efficacy of this methodology in an endemic, but low-prevalence, environment on a large sample cohort. *Strongyloides* sp. real-time PCR was positive in 37 more specimens than were detected by microscopy, supporting previous studies demonstrating the low sensitivity of FEAC for *Strongyloides* detection [11,13,43,44]. All *Strongyloides* cultures were negative in this study; however, this may have been due to delays during transport, the chilling of faecal samples during shipment, and the low larval load in most samples [20], as demonstrated by the large number of microscopy negative results.

In the absence of a gold standard, it is not clear if all the positive real-time PCR results are true positives. Positive results were obtained from patient samples where the residence was not in an area of known transmission, or without supporting laboratory markers, such as eosinophilia, though the limited value of eosinophilia as a predictor for strongyloidiasis has been discussed in previous studies [45]. Based upon the limited clinical information that is available, it was not possible to determine if these patients were exposed in the past. The reported specificity of the primers employed, based upon sequence analysis of amplicons, has approached 100% in almost all of the previously published papers [46,47,48,49]. However, in a recent *Strongyloides* PCR-based survey of samples from Cambodia and Timor Leste, primer-dimers led to false-positive results [50]. Altering the forward primer sequence slightly led to an improvement in the performance of the PCR, based upon the assessments made on specimens from the Northern Territory of Australia (Holt D, personal communication). It is possible that a similar phenomenon affected the results from this study, and further investigation is warranted. 

Thirteen samples showing *C*_t_ values of less than 40 on initial PCR were negative or had a *C*_t_ >40 upon repeat testing; at least one of these were found to be true *S. stercoralis* infections by nested PCR and sequencing. Using sequencing and SSCP of the nested PCR products as a confirmatory test substantiated that at least 31 of the samples that were tested contained *S. stercoralis*. While the nested PCR primers also led to non-specific amplification, this does not exclude lower concentrations of *Strongyloides* DNA in the affected samples, with preferential non-specific amplification. Investigations into PCR efficiency have demonstrated preferential amplification for G-C-rich templates over those with a significant A-T composition [51]. In the case of *Strongyloides stercoralis,* the whole genome sequence has an A-T content of 78% [52].

In terms of the most appropriate *C*_t_ cut-off value for the RT-PCR, some studies have utilised a *C*_t_ cut-off of 35 cycles [50,53]. However, some specimens in this study with *Strongyloides* sp. detected by microscopy, and others with eosinophilia and/or reactive serology, had *C*_t_ values of over 35. Similarly, Sultana et al. found that the sensitivity of the same RT-PCR that was employed in this study was low in samples with a low larval load, with only 5/32 samples delivering positive results and all with *C*_t_ values between 36–38 [40]. While a *C*_t_ cut-off value of 35 may be too low to detect low larval burden, a higher *C*_t_ cut-off may include false-positive reactions [54].

The ability to speciate *Strongyloides* by nested PCR was limited as the primers targeted the hypervariable region IV of the 18S rRNA gene, a section of genome containing only four single nucleotide polymorphisms (SNP) [55]. The low specificity of these primers in a mixed specimen, such as faeces, limits its application in diagnostics, and may make it for suitable for use on cultured larvae. When sequenced, the products that confirmed the presence of the amplified DNA as *Strongyloides stercoralis* were identical, without the presence of SNPs. Interestingly, this was the case despite two extracts identified as *S. stercoralis* by sequencing demonstrating an extra band on SSCP analysis. Whether this reflects an increased resolution of SSCP for detection of *S. stercoralis* genotypes or the paucity of available genomic data for *Strongyloides* species, has not been determined and warrants further investigation. 

This study indicated that the currently employed methods for the detection of *S. stercoralis* in north Queensland (direct microscopy, FEAC, and Harada-Mori) were not able to detect the majority of cases of strongyloidiasis. Transport time and refrigeration, as lower temperatures kill larvae, limits the applicability of conventional culture techniques for the routine diagnosis of strongyloidiasis in this setting [56]. The use of the qPCR for the detection of *Strongyloides* DNA in stool samples increased the diagnostic sensitivity, and would assist with individual diagnoses, screening, and public health interventions to eradicate this neglected disease.

## Figures and Tables

**Table 1:** Characteristics of patients with faecal samples submitted for diagnostic testing that yielded positive, equivocal or low level amplification *Strongyloides* sp. 18S rRNA real-time PCR compared to confirmatory testing using a second nested *Strongyloides* sp. 18S rRNA PCR with SSCP and sequencing. Values in bold are positive/above the normal range; * same patient; ^ another specimen on the same patient was PCR negative; Eos: eosinophil; nd; not done; SSCP: single strand conformation polymorphism; NW: North West; NTH: North; CW: Central West.

Age (Years)	Gender	Place of Residence	Region	Real-time PCR	Mean *C*_t_ Value	Nested PCR	Sequence Identity/SSCP Homology	*Strongyloides* Serology	Eosinophil Count (×10^9^/L)	Parasites Detected by FEAC Microscopy
2	F	Doomadgee *	NW	Positive	18.77	Detected	*S. stercoralis*	nd	nd	*S. stercoralis*
2	F	Doomadgee *	NW	Positive	19.16	Detected	*S. stercoralis*	nd	0.57	*S. stercoralis*
51	F	Doomadgee	NW	Positive	23.72	Detected	*S. stercoralis*	Reactive (3.1)	0.43	*S. stercoralis*
41	F	Doomadgee	NW	Positive	25.66	Detected	*S. stercoralis*	Reactive (1.6)	0.93	
2	F	Doomadgee *	NW	Positive	31.13	Detected	*S. stercoralis*	nd	3.69	*S. stercoralis*
9	M	Doomadgee	NW	Positive	35.25	Detected	*H. nana*	nd	2.84	*S. stercoralis*, *H. nana*, *B. hominis*, *E. nana*
70	M	Aurukun *	NW	Positive	27.44	Detected	*S. stercoralis*	nd	0.89	*B. hominis*
70	M	Aurukun *	NW	Positive	28.00	Detected	*S. stercoralis*	nd	0.72	*B. hominis*
28	M	Mount Isa	NW	Positive	28.10	Detected	*S. stercoralis*	nd	nd	
66	M	Mount Isa	NW	Positive	36.27	Detected	*B. hominis*	nd	0.02	*B. hominis*
30	M	Mount Isa	NW	Positive	36.41	Detected	*S. stercoralis*	nd	0.41	
61	M	Magnetic Island	NTH	Positive	31.15	Detected	*S. stercoralis*	nd	nd	
28	M	Magnetic Island	NTH	Positive	35.69	Detected	*S. stercoralis*	nd	0.30	
64	F	Magnetic Island	NTH	Positive	35.93	Detected	Human gene	nd	0.60	
51	F	Magnetic Island	NTH	Positive	36.12	Detected	*S. stercoralis*	nd	nd	*B. hominis*
36	M	Townsville	NTH	Positive	31.86	Detected	*S. stercoralis*	Reactive (4.0)	1.27	*B. hominis*
70	M	Townsville	NTH	Positive	34.12	Detected	*S. stercoralis*	nd	0.13	
40	M	Townsville	NTH	Positive	36.10	Detected	*S. stercoralis*	Reactive (3.1)	1.48	*H. nana*, *B. hominis*, *Entamoeba coli*
<1	F	Townsville	NTH	Positive	36.13	Detected	*S. stercoralis*	nd	0.00	
58	M	Townsville	NTH	Positive	36.57	Detected	*S. stercoralis*	nd	0.10	
<1	M	Townsville	NTH	Positive	37.08	Detected	*S. stercoralis*	nd	nd	
14	F	Townsville	NTH	Positive	37.31	Detected	*S. stercoralis*	nd	nd	
39	F	Townsville	NTH	Positive	38.51	Not Detected		nd	0.08	
69	F	Townsville	NTH	Positive	38.97	Detected	*Candida* sp.	nd	0.46	
51	F	Townsville	NTH	Positive	39.33	Detected	*Candida* sp.	nd	0.20	
43	F	Jundah	CW	Positive	34.32	Not Detected		nd	0.04	*B. hominis*
67	F	Mornington Island	NW	Positive	35.08	Detected	*B. hominis*	Reactive (4.1)	2.14	
1	M	Mornington Island	NW	Positive	36.96	Detected	Human gene	nd	8.04	*G. intestinalis*
35	M	Normanton	NW	Positive	35.81	Detected	*S. stercoralis*	nd	1.90	
36	M	Palm Island	NTH	Positive	36.53	Detected	*S. stercoralis*	nd	0.25	
<1 y	M	Palm Island	NTH	Positive	38.37	Not Detected		nd	nd	
1	M	Palm Island ^	NTH	Positive	38.48	Detected	Human gene	nd	nd	
20	F	Palm Island	NTH	Positive	38.84	Detected	*S. stercoralis*	nd	0.62	
78	M	Ayr	NTH	Positive	36.63	Detected	*S. stercoralis*	nd	0.14	
1	M	Cloncurry	NW	Positive	36.81	Not Detected		nd	nd	*B. hominis, E. nana*
94	F	Home Hill	NTH	Positive	36.95	Not Detected		nd	0.12	*B. hominis*
80	F	Greenvale	NTH	Positive	37.01	Detected	*S. stercoralis*	nd	0.09	
11	F	Charters Towers	NTH	Positive	37.15	Detected	*S. stercoralis*	nd	nd	
79	M	Hughenden	NW	Positive	37.66	Detected	*Saccharomyces* sp.	nd	1.05	
27	M	Camooweal	NW	Positive	38.12	Detected	*Saccharomyces* sp.	nd	0.25	*G. intestinalis*
84	M	Ingham	NTH	Positive	38.40	Not Detected		nd	0.09	*B. hominis*
63	F	Ingham	NTH	Positive	38.53	Not Detected		nd	0.09	
